# Supplementary material for: A Systematic Review of Research on Non-Maternal Caregivers’ Feeding of Children 0–3 Years
Source: Int J Environ Res Public Health. 2022 Nov 4;19(21):14463. doi: 10.3390/ijerph192114463 (PMC9658782; doi:10.3390/ijerph192114463)
Supplement: Supplementary file 1 [file ijerph-19-14463-s001.zip › Systematic review Supplementary Table S1.pdf]

Table S1. Index of information in the review meeting PRISMA Guidelines

**Section**	**#**	**Checklist item**	**Reported on page #**
Title	1	Identify the study as a systematic review, meta-analysis, or both.	1
Abstract: structured summary	2	Provide a structured summary including, as applicable: background; objectives; data sources; study eligibility, participants, and interventions; study appraisal and synthesis methods; results; limitations; conclusions and implications of key findings; systematic review registration number.	2
Introduction: rationale	3	Describe the rationale for the review in the context of what is already known.	3
Introduction: objectives	4	Provide an explicit statement of questions being addressed with reference to participants, interventions, comparisons, outcomes, and study design.	3
Methods			
Protocol and registration	5	Indicate if a review protocol exists and where it can be assessed.	NA
Eligibility criteria	6	Specific study characteristics used as criteria for eligibility.	3-4
Information sources	7	Describe information sources in the search and the date last searched.	3
Search	8	Present full electronic search strategy, such that it could be repeated.	3-4, Additional File 2

Study selection	9	State the process for selecting studies.	4
Data collection process	10	Describe method of data extraction from reports and any processes for obtaining and confirming data from investigators.	4-5
Data items	11	List and define all variables for which data were sought and any assumptions and simplifications made.	4-5
Risk of bias in individual studies	12	Describe methods used for assessing risk of bias of individual studies and how this information will be used in any data synthesis.	4-5
Summary measures	13	State the summary measures.	4-5
Synthesis of measures	14	Describe the methods of handling data and combining results of studies, if done, including measures of consistency for each meta-analysis.	4-5
Risk of bias across studies	15	Specify any assessment of risk of bias that may affect the cumulative evidence.	4-5
Additional analyses	16	Describe methods of additional analyses, if done.	NA
Results			
Study selection	17	Give numbers of studies screened, assessed for eligibility, and included in the review, with reason for exclusions at each stage, ideally with a flow diagram.	Figure 1

Study characteristics	18	For each study, present characteristics for which data were extracted and provide the citations.	28-30 (Table 1)
Risk of bias within studies	19	Present data on risk of bias of each study and if available, any outcome level assessment (see item 12).	Additional Files 3-4
Results of individual studies	20	For all outcomes considered, present for each study simple summary data for each intervention group and effect estimates and confidence intervals, ideally with a forest plot.	5-14, 28-30, Additional File 5
Synthesis of results	21	Present results of each meta-analysis done.	NA
Risk of bias across studies	22	Present results of any assessment of risk of bias across studies (see item 15).	4-5, 14-15
Additional analysis	23	Give results of additional analyses, if done.	NA
Discussion			
Summary of evidence	24	Summarize the main findings including the strength of evidence for each main outcome.	15-18
Limitations	25	Discuss limitations at study and outcome level and at review level.	18-21
Conclusions	26	Provide a general interpretation of the results in the context of other evidence, and implications for future research.	18-21

Funding	27		22
