# Supplementary material for: A Systematic Review of Research on Non-Maternal Caregivers’ Feeding of Children 0–3 Years
Source: Int J Environ Res Public Health. 2022 Nov 4;19(21):14463. doi: 10.3390/ijerph192114463 (PMC9658782; doi:10.3390/ijerph192114463)
Supplement: Supplementary file 1 [file ijerph-19-14463-s001.zip › Systematic review Supplementary Table S2.pdf]

Table S2: Search string for PubMed

(((infant) Mesh OR ("0-3 years Old") OR (child, preschool) OR (infant, newborn)) AND ((Grandparent) OR ("Non-Maternal Caregiver") OR (Babysitter) OR (Sibling) OR (Friend) OR (Father) Mesh OR ("Early Childhood Educator") OR ("childcare provider") OR ("child care provider") OR (non-parental) OR ("non-parent caregiver")) AND ((feeding) OR ("child care") OR ("infant care") OR ("feeding practices") OR ("infant feeding") OR (nutrition) OR ("feeding styles")) AND (("infant growth") OR (BMI) OR ("growth trajectory") OR (weight)))
