# Supplementary material for: A Systematic Review of Research on Non-Maternal Caregivers’ Feeding of Children 0–3 Years
Source: Int J Environ Res Public Health. 2022 Nov 4;19(21):14463. doi: 10.3390/ijerph192114463 (PMC9658782; doi:10.3390/ijerph192114463)
Supplement: Supplementary file 1 [file ijerph-19-14463-s001.zip › Systematic review Supplementary Table S3.pdf]

Table S3: Evaluation of design and methods for quantitative studies in the review

Study	Designed with research Q in mind	Measures allowed research Q to be answered clearly	Population clearly described	Study carried out well	Sample representative	Response rate reported	Population denominator reported	Measures valid and reliable	Appropriate measures for Qs	Same method for different groups	Method introduced bias	Study large enough	Adequate description of data	Appropriate statistical tests	Overall assessment
Ansuya et al. (2018)	To an extent	Yes	Yes	Yes	Yes	Yes	Yes	Yes	Yes	Yes	No	Yes	Yes	Yes	Good
Barrett, Wasser, Thompson, & Bentley (2018)	Yes	Yes	Yes	Yes	Yes	Yes	No	To an extent	Yes	Yes	No	To an extent	Yes	Yes	Fair
Benjamin-Neelon, & Neelon (2020)	Yes	Yes	Yes	Yes	Yes	Yes	No	To an extent	Yes	No	No	To an extent	Yes	Yes	Fair
Blaine et al. (2015)	Yes	Yes	Yes	Yes	Yes	No	No	To an extent	Yes	To an extent	No	To an extent	Yes	Yes	Fair
Blissett, Meyer, & Haycraft (2006)	Yes	Yes	Yes	Yes	Yes	No	No	Yes	Yes	Yes	No	To an extent	Yes	Yes	Fair
Chakona (2020)	Yes	Yes	Yes	Yes	Yes	No	No	Yes	Yes	Yes	No	To an extent	Yes	Yes	Fair
Chung et al. (2020)	No	Yes	Yes	Yes	Yes	Yes	Yes	Yes	Yes	Yes	No	Yes	Yes	Yes	Good
Dev, McBride, Speirs, Donovan, &	Yes	Yes	Yes	Yes	Yes	Yes	No	Yes	Yes	Yes	No	To an extent	Yes	Yes	Fair

Cho (2014)															
Freedman & Alvarez (2010)	Yes	Yes	Yes	Yes	To an extent	Yes	No	Yes	Yes	Yes	No	To an extent	Yes	Yes	Fair
Guerrero, Chu, Franke, & Kuo (2016)	Yes	Yes	Yes	Yes	Yes	No	No	Yes	Yes	Yes	No	Yes	Yes	Yes	Good
He, Li, & Wang (2018)	Yes	Yes	Yes	Yes	Yes	No	No	Yes	Yes	Yes	No	Yes	Yes	Yes	Good
Horodynski, Hoerr, & Coleman (2004)	Yes	Yes	Yes	Yes	Yes	Yes	No	To an extent	Yes	Yes	No	To an extent	Yes	Yes	Fair
Karmacharya, Cunningham, Choufani, & Kadiyala (2017)	Yes	Yes	Yes	Yes	Yes	No	No	Yes	Yes	Yes	No	Yes	Yes	Yes	Good
Katzow, Messito, Mendelsohn, Scott, & Gross (2021)	Yes	Yes	Yes	Yes	Yes	No	No	Yes	Yes	Yes	No	Yes	Yes	Yes	Good
Lanigan (2012)	Yes	Yes	Yes	Yes	Yes	No	No	Yes	Yes	Yes	No	To an extent	Yes	Yes	Fair
Mallan et al. (2013)	Yes	Yes	Yes	Yes	Yes	No	No	Yes	Yes	Yes	No	Yes	Yes	Yes	Good
Mallan et al. (2014)	Yes	Yes	Yes	Yes	Yes	No	No	Yes	Yes	Yes	No	Yes	Yes	Yes	Good
Metbulut,	Yes	Yes	Yes	Yes	To an extent	No	No	Yes	Yes	No	No	Yes	Yes	Yes	Good

Özmert, Teksam, & Yurdakök (2018)															
Reisz et al. (2019)	Yes	Yes	Yes	Yes	Yes	No	No	Yes	Yes	Yes	No	To an extent	Yes	Yes	Fair
Sigman-Grant et al. (2011)	Yes	Yes	Yes	Yes	Yes	No	Yes	Yes	Yes	Yes	No	Yes	Yes	Yes	Good
Tan et al. (2019)	Yes	Yes	Yes	Yes	Yes	Yes	Yes	To an extent	Yes	Yes	No	Yes	Yes	Yes	Good
Tovar et al. (2019)	Yes	Tes	Yes	Yes	Yes	Yes	No	Yes	Yes	Yes	No	Yes	Yes	Yes	Good
Wasser et al. (2013)	Yes	Yes	Yes	Yes	Yes	No	No	Yes	Yes	Yes	No	Yes	Yes	Yes	Good
Yue et al. (2018)	Yes	Yes	Yes	Yes	Yes	No	No	Yes	Yes	Yes	No	Yes	Yes	Yes	Good
Zhang et al. (2018)	Yes	Yes	Yes	Yes	Yes	Yes	No	Yes	Yes	Yes	No	Yes	Yes	Yes	Good
