# Supplementary material for: A Systematic Review of Research on Non-Maternal Caregivers’ Feeding of Children 0–3 Years
Source: Int J Environ Res Public Health. 2022 Nov 4;19(21):14463. doi: 10.3390/ijerph192114463 (PMC9658782; doi:10.3390/ijerph192114463)
Supplement: Supplementary file 1 [file ijerph-19-14463-s001.zip › Systematic review Supplementary Table S4.pdf]

Table S4: Evaluation of design and methods for qualitative studies in the review

Study	Credible findings, knowledge extended	Addressed well original aims and purpose, and scope for wider inference	Clear basis for evaluative appraisal	Defensible research design	Well defended sample	Described well sample composition and coverage	Carried out well data collection	Conveyed well approach to analysis	Portrayed well contexts of data sources	Explored well diversity of content/ perspective, Conveyed well richness of data	Clear links bw data and conclusions	Clearly conveyed assumptions, values, theory	Attention to ethical issues	Clear reporting, Adequately documented research process	Overall assessment
Anderson, Nicklas, Spence, & Kavanagh (2010)	Yes, Yes	Yes, To an extent	Yes	Yes	Yes	Yes	Yes	Yes	To an extent	No, Yes	Yes	Yes	Yes	Yes, Yes	Fair
Chakona (2020)	Yes, Yes	Yes, Yes	Yes	Yes	Yes	Yes	Yes	Yes	Yes	Yes, Yes	Yes	Yes	Yes	Yes, Yes	Good
Eli, Hörnell, Etminan Malek, & Nowicka (2017)	Yes, Yes	Yes, Yes	Yes	Yes	Yes	Yes	Yes	Yes	To an extent	Yes, Yes	Yes	Yes	Yes	Yes, Yes	Good
Horodynski & Arndt (2005)	Yes, Yes	Yes, Yes	Yes	To an extent	To an extent	Yes	Yes	To an extent	To an extent	To an extent, To an extent	Yes	Yes	Yes	Yes, Yes	Fair
Hossain et al. (2018)	Yes, Yes	Yes, Yes	Yes	Yes	Yes	Yes	Yes	Yes	Yes	Yes, Yes	Yes	Yes	Yes	Yes, Yes	Good
Jiang et al. (2007)	Yes, Yes	Yes, Yes	Yes	Yes	Yes	Yes	Yes	Yes	To an extent	Yes, Yes	Yes	Yes	Yes	Yes, Yes	Good
Khandpur,	Yes, Yes	Yes, Yes	Yes	Yes	Yes	Yes	Yes	Yes	To an extent	Yes, Yes	Yes	Yes	Yes	Yes, Yes	Good

Charles, & Davison (2016)															
Lidgate, Li, & Lindenmeyer (2018)	Yes, Yes	Yes, Yes	Yes	Yes	To an extent	Yes	Yes	Yes	Yes	Yes, Yes	Yes	Yes	Yes	Yes, Yes	Good
Lindsay et al. (2020)	Yes, Yes	Yes, Yes	Yes	Yes	Yes	Yes	Yes	Yes	Yes	Yes, Yes	Yes	Yes	Yes	Yes, Yes	Good
Love et al. (2020)	Yes, Yes	Yes, Yes	Yes	Yes	Yes	Yes	Yes	Yes	Yes	Yes, Yes	Yes	Yes	Yes	Yes, Yes	Good
Rachmi, Hunter, Li, & Baur (2017)	Yes, Yes	Yes, Yes	Yes	Yes	Yes	Yes	Yes	Yes	Yes	Yes, Yes	Yes	Yes	Yes	Yes, Yes	Good
Roshita, Schubert, & Whittaker (2012)	Yes, Yes	Yes, Yes	Yes	Yes	Yes	Yes	Yes	Yes	Yes	Yes, Yes	Yes	Yes	Yes	Yes, Yes	Good
Tan et al. (2019)	Yes, Yes	Yes, Yes	Yes	Yes	Yes	To an extent	Yes	Yes	To an extent	To an extent, To an extent	Yes	Yes	Yes	Yes, Yes	Fair
Vandeweghe et al. (2016)	Yes, Yes	Yes, Yes	Yes	To an extent	Yes	Yes	Yes	Yes	To an extent	Yes, Yes	Yes	Yes	Yes	Yes, Yes	Fair
Wallace, Lombardi, De Backer, Costello, & Devine (2020)	Yes, Yes	Yes, Yes	Yes	Yes	Yes	Yes	Yes	Yes	Yes	Yes, Yes	Yes	Yes	Yes	Yes, Yes	Good
